# Radiographic Changes in Patellar Height Following Rapid Weight Loss After Sleeve Gastrectomy: A Retrospective Observational Study

**DOI:** 10.3390/jcm15187137

**Published:** 2026-09-14

**Authors:** Emre Erdoğan, Elif Nur Gencer, Furkan Türkoğlu, Ahmet Çulcu, Soner Sarı, Özgür Doğan

**Affiliations:** 1 Department of General Surgery, Aktif International Hospital, 41275 Kocaeli, Türkiye; genelcerrahemreerdogan@gmail.com; 2 Department of General Surgery, Tuzla State Hospital, 34947 İstanbul, Türkiye; elifnurkocmar@yahoo.com; 3 Department of Orthopaedics and Traumatology, Sakarya Sadıka Sabancı State Hospital, 54580 Sakarya, Türkiye; dr.ahmetculcu@gmail.com; 4 Department of Orthopaedics and Traumatology, Ankara Bilkent City Hospital, 06800 Ankara, Türkiye; sarisnr97@gmail.com (S.S.); dr.ozgurdogan@gmail.com (Ö.D.)

**Keywords:** sleeve gastrectomy, obesity, patellar height, knee biomechanics, mechanical unloading, retrospective observational study, single-center study

## Abstract

**Background/Objectives**: Rapid weight loss following sleeve gastrectomy substantially reduces mechanical loading across the lower extremities, yet its effects on patellar height remain unknown. This study investigated early postoperative radiographic changes in patellar height following sleeve gastrectomy and evaluated whether these changes were associated with the magnitude of weight loss or body mass index (BMI) reduction. **Methods**: This retrospective observational study was conducted at a specialized high-volume bariatric surgery center in Kocaeli, Türkiye. Standardized bilateral knee radiographs were obtained preoperatively and at the routine fourth postoperative month in patients undergoing primary sleeve gastrectomy. Patellar height was assessed using the Insall–Salvati (IS), Blackburne–Peel (BP), and Caton–Deschamps (CD) indices. **Results**: A total of 155 consecutive patients were included. Body weight and BMI decreased significantly by the fourth postoperative month (both *p* < 0.001). The IS index remained unchanged (1.13 [IQR, 0.25] vs. 1.11 [IQR, 0.22]; *p* = 0.314), whereas the BP index decreased from 0.94 [IQR, 0.26] to 0.90 [IQR, 0.23] (*p* = 0.004), and the CD index decreased from 1.06 [IQR, 0.20] to 1.04 [IQR, 0.32] (*p* = 0.039). Although absolute postoperative BP values demonstrated weak associations with baseline and postoperative body weight and BMI, the magnitude of postoperative changes (Δ values) was not independently associated with the extent of weight loss or BMI reduction. In contrast, baseline patellar height independently predicted postoperative radiographic adaptation. **Conclusions**: Rapid postoperative weight loss following sleeve gastrectomy was associated with early radiographic changes in patellar height. Although these changes were statistically significant, they were modest in magnitude and appeared to be influenced predominantly by baseline patellar anatomy, which independently predicted postoperative radiographic adaptation, rather than by the extent of weight loss.

## 1. Introduction

Obesity is now recognized as a complex chronic disease requiring a comprehensive, multidisciplinary treatment strategy that includes lifestyle modifications, nutritional support, pharmacological therapy, bariatric surgery, and long-term follow-up [1]. Recent advances in anti-obesity medications, particularly glucagon-like peptide-1 receptor agonists such as semaglutide, have substantially expanded the therapeutic possibilities and demonstrated substantial weight loss and cardiometabolic benefits in clinical trials and real-world practice [2,3,4]. Nevertheless, bariatric surgery remains the most effective intervention for rapidly and durably reducing weight in patients with severe obesity, and it continues to play a central role in modern obesity management [1,4,5].

Increased body mass imposes excessive mechanical loading on the lower extremities and may alter joint biomechanics, gait patterns, and load distribution, thereby contributing to progressive structural and functional deterioration [6]. Consequently, obesity has been consistently associated with an increased prevalence of musculoskeletal pain and degenerative joint disease, including knee and hip osteoarthritis, and these conditions may substantially impair mobility, physical function, and quality of life [6,7,8]. By markedly reducing body weight and lower-extremity loading, bariatric surgery has been shown to improve joint pain, physical function, and patient-reported outcomes, while longer-term studies have also demonstrated sustained reductions in osteoarthritis-related symptoms and further improvements in functional outcomes [9,10].

Sleeve gastrectomy is a restrictive bariatric procedure in which a substantial portion of the stomach is surgically removed, leaving a narrow, tubular gastric reservoir and resulting in substantial postoperative weight loss [11,12]. Although sleeve gastrectomy is well recognized for its long-term effectiveness in obesity treatment, the early postoperative period is characterized by rapid weight loss accompanied by metabolic improvement and marked reduction in lower-extremity mechanical loading [6,12,13,14]. Although chronic obesity-related structural degeneration may not be fully reversible, the first postoperative months provide a unique opportunity to investigate the earliest biomechanical consequences of rapid mechanical unloading. The first 3–6 months following sleeve gastrectomy are characterized by the most pronounced reductions in body weight and lower-extremity loading. During this period, previous studies have demonstrated significant improvements in musculoskeletal function, including reductions in knee and low back pain as early as three months after surgery [15] and improvements in gait parameters within the first six postoperative months [16]. Moreover, experimental evidence suggests that musculoskeletal soft tissues, including tendons, require several weeks to months to adapt to altered mechanical loading, indicating that this early postoperative phase is biologically relevant for detecting structural adaptation [17]. Accordingly, measurable biomechanical changes may be expected during this period before longer-term remodeling occurs. Recent studies demonstrated early improvement in Meary’s angle together with a reduction in the intermetatarsal angle after sleeve gastrectomy, suggesting that selected radiographic alignment parameters rapidly respond to postoperative mechanical unloading [18,19]. These findings suggest that rapid postoperative weight loss may induce early biomechanical adaptations within the lower extremity. However, whether similar adaptations also occur at the level of the knee and influence radiographic patellar height remains unknown. To the best of our knowledge, no previous study has specifically investigated the early effects of bariatric surgery-induced weight loss on radiographic patellar height.

Patellar height is an important component of the knee extensor mechanism and contributes substantially to patellofemoral biomechanics and knee stability [20,21]. Patella alta (superior patellar position) has been particularly associated with an increased susceptibility to patellofemoral instability, whereas patella baja (inferior patellar position) may be associated with restricted knee motion, stiffness, and altered extensor mechanism function [22,23]. In patients with severe obesity, alterations in lower-extremity alignment, including changes in the quadriceps angle, may further modify patellofemoral biomechanics and extensor mechanism function. Although Salem et al. [24] identified BMI as a factor associated with the Insall-Salvati index, this relationship is thought to reflect biomechanical rather than intrinsic anatomical mechanisms. Given its important role in patellofemoral biomechanics, patellar height has therefore been investigated in various clinical settings, including total knee arthroplasty, high tibial osteotomy, meniscal pathology, anterior cruciate ligament injury and reconstruction, and periarticular fractures [24,25,26,27]. Although patellar height is influenced by multiple anatomical and biomechanical factors, the effect of rapid postoperative mechanical unloading has not been adequately investigated. In particular, it remains unclear whether the substantial mechanical unloading following sleeve gastrectomy is accompanied by an adaptive change in patellar position or sagittal patellofemoral balance.

Therefore, the primary objective of the present study was to investigate whether rapid weight loss following sleeve gastrectomy is associated with early radiographic changes in patellar height. Secondary objectives were to compare complementary patellar height indices and to determine whether postoperative adaptation was associated with weight loss, BMI reduction, or baseline patellar height. We hypothesized that rapid postoperative unloading would induce measurable radiographic adaptation of the knee extensor mechanism.

## 2. Materials and Methods

### 2.1. Study Design and Ethical Approval

This single-center retrospective observational cohort study was conducted using prospectively collected data from the bariatric surgery database of a specialized high-volume bariatric surgery center. All patients included in the study had been followed prospectively according to the institution’s standardized bariatric surgery follow-up protocol; however, the present study was designed as a retrospective analysis of these prospectively collected data. The study was designed, conducted, and reported in accordance with the Strengthening the Reporting of Observational Studies in Epidemiology (STROBE) Statement [28]. A completed STROBE checklist has been provided as Supplementary Material. The study protocol was reviewed and approved by the Scientific and Ethical Review Board for Medical Research of Ankara Bilkent City Hospital (Approval No: TABED 2-26-2345; Date: 10 June 2026). All procedures were performed in accordance with the ethical principles of the Declaration of Helsinki and its subsequent amendments. During the preparation of this manuscript, ChatGPT (GPT-5.5; OpenAI, San Francisco, CA, USA) was used exclusively for minor language editing, including corrections of spelling, grammar, punctuation, and stylistic clarity. The artificial intelligence tool was not used for the conception, design, conduct, analysis, interpretation, or reporting of the study, nor did it contribute to the scientific content, results, or conclusions. All intellectual content, methodological decisions, and final manuscript revisions remained the sole responsibility of the authors.

### 2.2. Patient Selection

Between November 2025 and April 2026, consecutive patients who underwent primary laparoscopic sleeve gastrectomy performed by a single senior bariatric surgeon (E.E.) were retrospectively screened for eligibility from the institutional database after Institutional Review Board approval. All clinical and radiographic data analyzed in the present study had been acquired previously as part of routine preoperative evaluation and standardized postoperative follow-up, independent of the present study. No study-specific imaging, data collection, or patient follow-up was performed before ethics approval, and the study did not alter routine patient management or imaging acquisition. All procedures were performed using a standardized surgical technique consistent with current bariatric surgical practice [18,29].

At the study institution, musculoskeletal assessment constitutes a routine component of the preoperative evaluation and postoperative follow-up of patients undergoing bariatric surgery. Accordingly, standardized standing knee radiographs are routinely obtained preoperatively and during routine postoperative follow-up to facilitate the early identification of musculoskeletal abnormalities, monitor biomechanical adaptations, and ensure consistent longitudinal radiographic assessment. The routine fourth postoperative month evaluation was selected for the present study because it corresponds to the period of rapid postoperative weight loss and represents the first standardized radiographic follow-up after surgery, allowing the assessment of the early biological adaptation of musculoskeletal soft tissues to altered mechanical loading.

Patients were eligible for inclusion if they:

- underwent primary laparoscopic sleeve gastrectomy during the study period;
- were 18 years of age or older;
- completed the institution’s standardized bariatric follow-up protocol;
- had available standardized standing bilateral knee radiographs obtained both preoperatively and at the routine fourth postoperative month; and
- had complete demographic, anthropometric, and radiographic data available for analysis.

Patients were excluded if they had a previous history of fracture or surgical intervention involving either knee, congenital or acquired structural abnormalities affecting radiographic patellar height measurements, advanced radiographic knee osteoarthritis (Kellgren–Lawrence grade III or IV), incomplete postoperative follow-up, bariatric procedures other than primary sleeve gastrectomy, revision bariatric surgery, or combined bariatric surgical procedures. All structural knee abnormalities and osteoarthritic changes were identified during the routine preoperative clinical and radiographic assessment.

Apart from these predefined exclusion criteria, all consecutive eligible patients who completed the standardized fourth postoperative follow-up were included without additional sampling or selection to minimize selection bias. No formal sample size calculation was performed because all consecutive eligible patients treated during the predefined study period were included in the final analysis. No missing data were present in the final dataset, as only patients with complete demographic, anthropometric, and radiographic data were included in the final analysis.

### 2.3. Clinical and Demographic Data Collection

Demographic and anthropometric data were retrieved from the prospectively maintained institutional database. The recorded variables included age, sex, height (m), body weight (kg), and body mass index (BMI, kg/m^2^). Postoperative body weight and BMI were obtained at the routine 4-month postoperative follow-up visit.

Changes in body weight (ΔWeight) and BMI (ΔBMI) were calculated for each patient and included in the statistical analyses. Δ values were calculated by subtracting the postoperative value from the corresponding preoperative value. Accordingly, positive Δ values indicated a postoperative decrease, whereas negative Δ values indicated a postoperative increase.

### 2.4. Radiographic Assessment

Standing true lateral radiographs of both knees were obtained according to the institutional musculoskeletal imaging protocol using the same radiographic equipment. Radiographs were acquired with the knee positioned at approximately 30° of flexion and the X-ray beam centered on the knee joint, 1.5–2.0 cm distal to the apex of the patella, by experienced radiology technicians following standardized patient-positioning procedures. Before radiographic measurements were performed, the orthopedic review panel verified the technical adequacy of all radiographs, including true lateral positioning and overall image quality, and no radiographs were excluded because of inadequate positioning or image quality. The same institutional imaging protocol was applied for both the preoperative and postoperative examinations to ensure consistency between serial radiographic assessments. Because rapid postoperative weight loss represents a systemic intervention affecting both lower extremities, and to avoid statistical dependence between paired observations, the right knee was selected a priori for all participants according to the study protocol. No randomization procedure was used, as the same anatomical side was analyzed consistently in every patient. Furthermore, this study was designed to evaluate systemic radiographic adaptation rather than side-specific symptoms or unilateral pathology.

Radiographic patellar height was evaluated digitally using the institutional Picture Archiving and Communication System (PACS; Sarus PACS, Teknoritma Yazılım Hizmetleri A.Ş., Ankara, Türkiye; https://www.saruspacs.com/) with three previously validated radiographic indices:

- The Insall–Salvati (IS) index was calculated as the ratio of patellar tendon length to the greatest diagonal length of the patella.
- The Blackburne–Peel (BP) index was calculated as the ratio between the perpendicular distance from the inferior margin of the patellar articular surface to the tibial plateau line and the length of the patellar articular surface.
- The Caton–Deschamps (CD) index was calculated as the ratio between the distance from the inferior margin of the patellar articular surface to the anterosuperior margin of the tibial plateau and the length of the patellar articular surface.

All measurements were performed according to the original descriptions of each method [30,31,32]. To minimize measurement variability and ensure consistent identification of anatomical landmarks, the preoperative and postoperative radiographs of each patient were evaluated side-by-side during the same assessment session.

### 2.5. Observer Assessment

All radiographic measurements were independently performed by a three-member orthopedic review panel consisting of two senior board-certified orthopedic surgeons (A.Ç. and S.S.) and one board-certified professor of orthopedic surgery (Ö.D.) with extensive clinical and academic experience in patellar height assessment and musculoskeletal radiographic research [20,22,25]. Each radiograph was reviewed simultaneously by the panel, and all measurements were established by consensus. Because no independent observer-specific measurements were generated, interobserver reliability statistics, such as the intraclass correlation coefficient, were not applicable.

All reviewers were blinded to the patients’ demographic and clinical characteristics, as well as the magnitude of postoperative weight loss and BMI reduction. All radiographic measurements were performed through a consensus-based assessment by the three-member review panel, and the resulting consensus values were used for all subsequent statistical analyses.

### 2.6. Primary and Secondary Outcomes

The primary outcome of the study was the change in radiographic patellar height between the preoperative assessment and the routine fourth postoperative month follow-up after sleeve gastrectomy. Changes in the Insall–Salvati, Blackburne–Peel, and Caton–Deschamps indices were compared between the two time points.

Secondary outcomes included the evaluation of the relationships between anthropometric parameters and radiographic patellar height. Correlation analyses were performed using both postoperative patellar height indices and their postoperative changes (Δ values) to determine whether postoperative body weight, BMI, and the magnitude of weight loss were associated with radiographic patellar height and its early postoperative changes. For all variables, Δ values were calculated as the preoperative value minus the postoperative value. Accordingly, positive Δ values indicated a postoperative decrease, whereas negative Δ values indicated a postoperative increase relative to baseline. In addition, the direction of postoperative change (increase, decrease, or no change) was evaluated descriptively for each patellar height index.

### 2.7. Statistical Analysis

Statistical analyses were performed using IBM SPSS Statistics for Windows (Version 26.0; IBM Corp., Armonk, NY, USA). The normality of continuous variables was assessed using the Shapiro–Wilk test, together with visual inspection of histograms and Q-Q plots. As all continuous variables demonstrated non-normal distributions, they were summarized as median (interquartile range [IQR]) and minimum–maximum values. Categorical variables were summarized as frequencies and percentages and were used for descriptive purposes only; therefore, no inferential statistical comparisons of categorical variables were performed. The direction of postoperative changes (increase, decrease, or no change) for each patellar height index was evaluated descriptively and is expressed as frequencies and percentages. All statistical tests were two-sided, and a *p* value < 0.05 was considered statistically significant.

Preoperative and postoperative anthropometric and radiographic measurements were compared using the Wilcoxon signed-rank test. Correlation analyses between anthropometric variables and radiographic patellar height indices, including both postoperative values and postoperative changes (Δ values), were performed using Spearman’s rank correlation coefficient (r). Positive r values indicate positive correlations, whereas negative r values indicate inverse correlations. Correlation strength was interpreted according to the absolute value of r as very weak (0.00–0.19), weak (0.20–0.39), moderate (0.40–0.59), strong (0.60–0.79), or very strong (0.80–1.00). Because the correlation analyses were prespecified exploratory analyses intended to evaluate potential associations rather than to test multiple independent primary hypotheses, no formal adjustment for multiple comparisons was applied. Accordingly, correlation findings should be interpreted as exploratory.

To further evaluate whether baseline patellar height independently predicted postoperative radiographic changes after adjustment for potential confounders, separate multiple linear regression analyses were performed for ΔIS, ΔBP, and ΔCD. Each model included the corresponding preoperative patellar height index, age, sex, preoperative BMI, and BMI reduction (ΔBMI) as independent variables. Preoperative body weight and weight loss (ΔWeight) were not entered into the regression models because of their mathematical collinearity with BMI-derived variables, thereby minimizing the risk of multicollinearity.

## 3. Results

### 3.1. Patient Characteristics and Radiographic Findings

**Figure 1:**
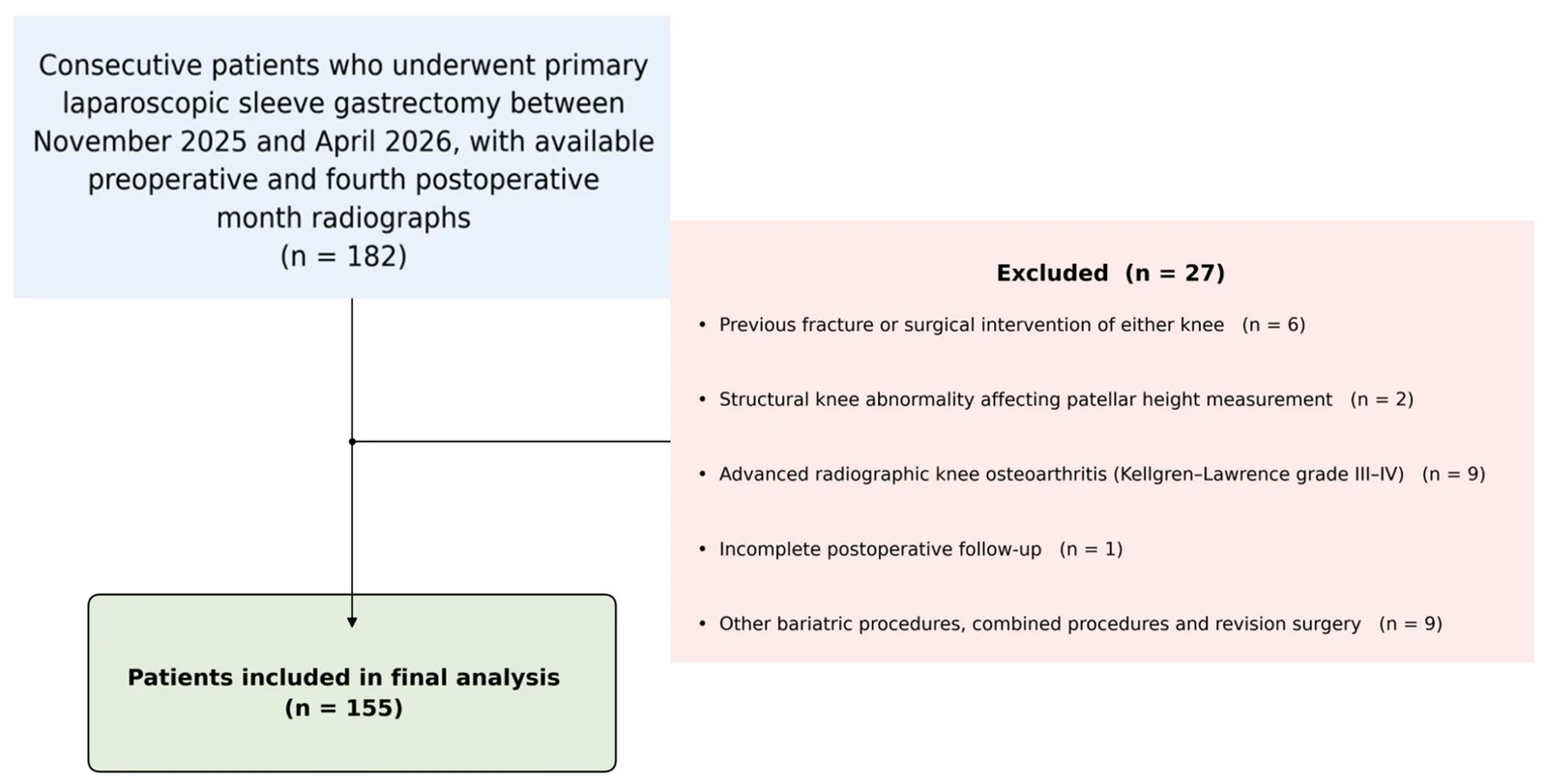
Study flow diagram illustrating patient selection, the application of the inclusion and exclusion criteria, and the final study cohort included in the statistical analyses.

The patient selection process is illustrated in Figure 1. A total of 155 consecutive patients were included in the final analysis. Overall, substantial postoperative weight loss was accompanied by differential radiographic responses among the patellar height indices. While the IS index remained stable, the BP and CD indices demonstrated modest postoperative reductions. Detailed demographic, anthropometric, and radiographic findings are presented in Table 1.

### 3.2. Distribution of Postoperative Changes in Patellar Height Indices

**Figure 2:**
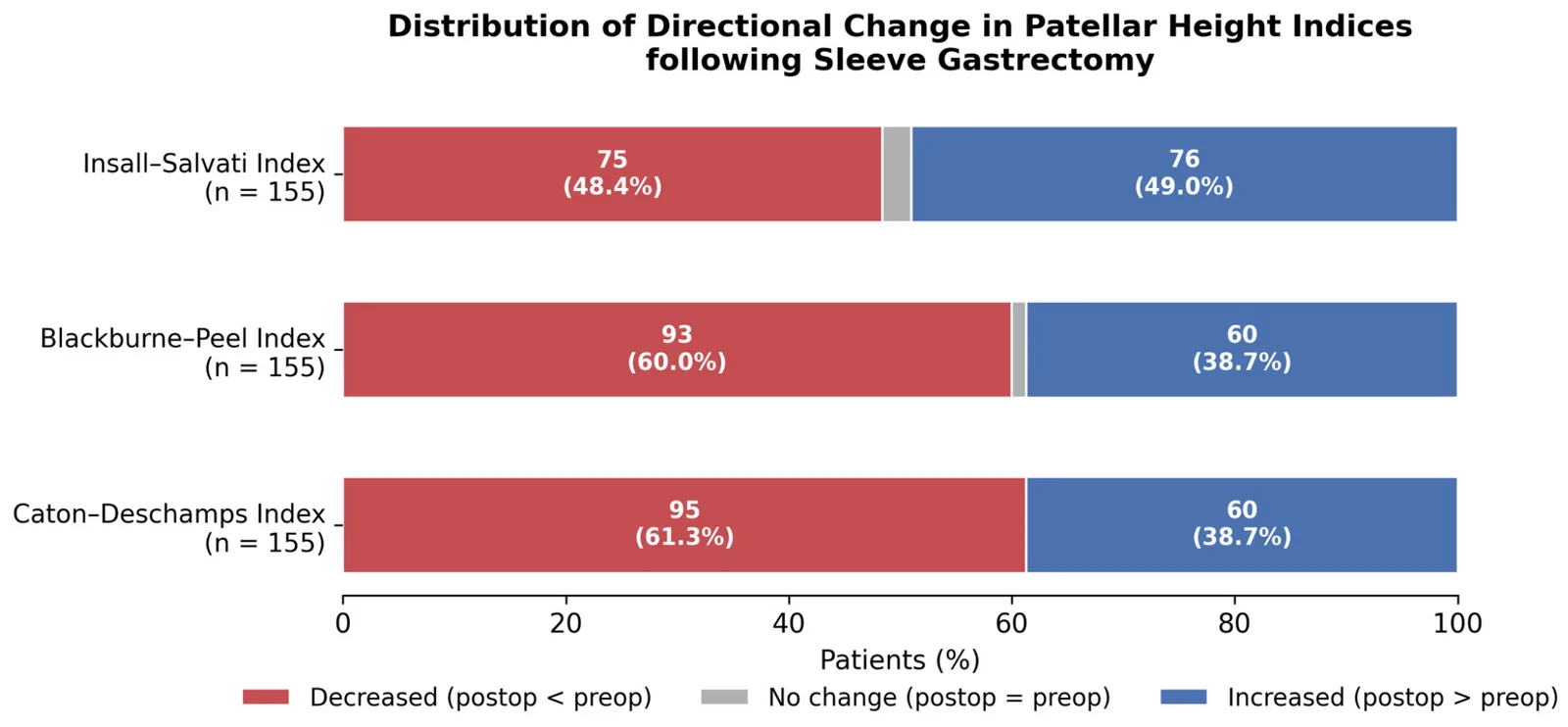
Direction of postoperative changes in radiographic patellar height indices following sleeve gastrectomy. Bars represent the proportions of patients demonstrating decreased, unchanged, or increased postoperative values relative to preoperative measurements for the Insall–Salvati, Blackburne–Peel, and Caton–Deschamps indices.

The direction of postoperative changes differed among the evaluated patellar height indices (Figure 2). The IS index demonstrated a nearly symmetrical distribution, with comparable proportions of patients showing increased (76/155, 49.0%) and decreased (75/155, 48.4%) postoperative values, whereas only four patients (2.6%) showed no change. In contrast, decreases were more frequently observed for both the BP and CD indices. BP values decreased in 93 patients (60.0%), increased in 60 patients (38.7%), and remained unchanged in two patients (1.3%). Similarly, CD values decreased in 95 patients (61.3%), increased in 60 patients (38.7%), and remained unchanged in no patients. The complete distribution of directional changes is presented in Figure 2.

### 3.3. Correlation Analyses

Correlation analyses were performed using both the postoperative patellar height indices and their corresponding postoperative changes (Δ values). Among the postoperative measurements, the BP index, which demonstrated a significant postoperative reduction, showed weak positive correlations with preoperative body weight (r = 0.221; *p* = 0.006), preoperative BMI (r = 0.214; *p* = 0.007), postoperative body weight (r = 0.208; *p* = 0.009), and postoperative BMI (r = 0.223; *p* = 0.005). In contrast, the postoperative CD index was not significantly associated with any anthropometric parameter (all *p* > 0.050). Analysis of postoperative changes demonstrated that neither ΔBP nor ΔCD was significantly correlated with changes in body weight or BMI (all *p* > 0.050). However, both ΔBP and ΔCD showed weak-to-moderate positive correlations with their respective preoperative values (all *p* < 0.05), suggesting that baseline radiographic measurements were associated with the magnitude of postoperative change. Furthermore, ΔBP and ΔCD demonstrated a moderate positive correlation with each other (r = 0.541; *p* < 0.001), whereas ΔIS showed no significant correlation with either ΔBP or ΔCD. In contrast, ΔIS demonstrated statistically significant but very weak positive correlations with both Δkg (r = 0.190; *p* = 0.018) and ΔBMI (r = 0.184; *p* = 0.022). Detailed correlation analyses are presented in Table 2.

### 3.4. Multivariable Regression Analyses

To further evaluate whether baseline patellar height independently influenced postoperative radiographic adaptation, separate multiple linear regression analyses were performed for ΔIS, ΔBP, and ΔCD after adjustment for age, sex, preoperative BMI, and BMI reduction. Preoperative body weight and weight loss (ΔWeight) were not simultaneously included in the models because of their strong collinearity with BMI-derived variables, which could introduce multicollinearity and compromise model stability. Baseline IS (β = 0.216; *p* = 0.013) and baseline BP (β = 0.257; *p* = 0.002) remained independent predictors of their respective postoperative changes, whereas neither age, sex, and preoperative BMI nor BMI reduction was independently associated with postoperative radiographic adaptation. The regression model for ΔCD was not statistically significant (overall model, *p* = 0.099) (Table 3).

## 4. Discussion

In the present study, we investigated the early effects of rapid weight loss following sleeve gastrectomy on radiographic patellar height, an important component of knee extensor mechanism biomechanics. To our knowledge, this is the first study to examine whether the substantial mechanical unloading that occurs during the early postoperative period is accompanied by measurable alterations in radiographic patellar height. The present study demonstrated significant reductions in the BP and CD indices within the first four postoperative months, whereas the IS index remained unchanged. Although both BP and CD changed significantly, the magnitude of these changes was not associated with the extent of weight loss or BMI reduction. The strongest correlations observed in the present study were between baseline patellar height indices and their corresponding postoperative changes. Consistent with these findings, multivariable regression analyses demonstrated that baseline IS and BP indices independently predicted their respective postoperative changes after adjustment for age, sex, preoperative BMI, and BMI reduction, suggesting that baseline anatomical characteristics may influence the extent of early radiographic adaptation following bariatric surgery.

Contemporary obesity management has evolved from a surgical-only approach to a multidisciplinary treatment model integrating lifestyle modification, pharmacological therapy, and bariatric surgery, tailored to individual patient characteristics and treatment goals. Recent studies have demonstrated that glucagon-like peptide-1 receptor agonists, particularly semaglutide, can achieve significant weight loss and improve cardiometabolic health in randomized clinical trials and in real-world settings [1,2,3,4,5]. This expands the range of treatment options available to patients with obesity. However, bariatric surgery remains the most effective intervention for achieving rapid and long-lasting weight loss, especially for patients with severe obesity [5]. Despite these advances, the musculoskeletal consequences of substantial weight loss have received considerably less attention than metabolic outcomes. To our knowledge, no previous study has investigated whether rapid weight loss is accompanied by early radiographic changes in patellar height. Therefore, the present findings extend the current literature by providing objective, radiographic evidence that the biomechanical adaptation of the knee extensor mechanism may occur during the early postoperative period following sleeve gastrectomy. Although there are no comparable musculoskeletal imaging studies for patients who have achieved weight loss through GLP-1 receptor agonist therapy, the growing use of semaglutide and similar drugs highlights the need to determine if the biomechanical adaptations observed after bariatric surgery also occur with pharmacologically induced weight loss. Therefore, comparative longitudinal studies evaluating surgical and pharmacological weight-loss strategies may provide important insights into whether these musculoskeletal adaptations are primarily driven by mechanical unloading or procedure-specific physiological mechanisms.

Previous studies evaluating the effects of bariatric surgery on the knee have primarily focused on pain, functional outcomes, gait characteristics, and osteoarthritis-related changes rather than radiographic patellofemoral alignment [15,16,33,34]. The present study extends the existing literature by demonstrating that rapid postoperative weight loss is accompanied by early radiographic changes in patellar height, suggesting that the knee extensor mechanism also undergoes measurable adaptation during the early postoperative period. Although the observed reductions in the BP and CD indices were modest, they suggest that radiographic parameters reflecting the relationship between the patella and tibial plateau may be sensitive to the substantial mechanical unloading that occurs during the early postoperative period. Despite these significant postoperative changes, the magnitude of change in neither BP (ΔBP) nor CD (ΔCD) was associated with preoperative or postoperative body weight, BMI, or the magnitude of weight loss. Furthermore, postoperative CD values were not significantly associated with any anthropometric measurement, whereas postoperative BP demonstrated only weak positive correlations with body weight and BMI. These associations were limited to the absolute postoperative BP values and were not observed for the magnitude of postoperative change (ΔBP). Moreover, multivariable regression analysis demonstrated that baseline BP independently predicted ΔBP after adjustment for age, sex, preoperative BMI, and BMI reduction, whereas neither BMI nor BMI reduction independently influenced postoperative radiographic adaptation. Similarly, Li et al. [34] reported that, although weight loss improved knee range of motion and patient-reported outcomes, no significant alterations in gait kinematics were observed. Likewise, previous investigations have consistently demonstrated that improvements in pain and functional performance after bariatric surgery are often disproportionate to measurable structural or biomechanical changes, supporting the concept that early functional recovery and radiographic adaptation may follow partially independent trajectories [15,16,33]. Together, these findings suggest that early biomechanical adaptation may be related to the presence of rapid mechanical unloading itself rather than being directly proportional to the magnitude of weight reduction. One possible explanation is that the observed radiographic changes reflect an early adaptive response to reduced mechanical loading rather than a dose-dependent effect of weight loss. If this adaptive response is initiated soon after unloading, additional weight reduction during the early postoperative period may have only a limited influence on patellar height. Alternatively, individual anatomical characteristics and baseline extensor mechanism geometry may play a greater role in determining the extent of radiographic adaptation than the amount of weight lost alone, a hypothesis further supported by the multivariable regression analyses demonstrating baseline patellar height, rather than BMI-related variables, as the principal determinant of postoperative change. However, in the present study, we evaluated only the early postoperative period. Whether a dose-dependent relationship emerges over longer follow-up, as weight loss and BMI reduction continue to progress during the first postoperative year, remains unknown. Future longitudinal studies with extended follow-up are warranted to determine whether continued weight reduction results in additional alterations in patellar height and knee biomechanics.

Another important finding of the present study was the discordant behavior of the three patellar height indices. Although both BP and CD demonstrated significant postoperative reductions, the IS index remained unchanged throughout the study period. A similar dissociation between the IS index and the tibial plateau-referenced indices (BP and CD) has also been reported following high tibial osteotomy, where BP and CD changed significantly whereas IS remained largely unchanged, suggesting that these indices may reflect different aspects of knee biomechanics [35]. This discrepancy may be attributable to the different anatomical landmarks and biomechanical characteristics incorporated into each measurement method. Unlike BP and CD, which assess the position of the patellar articular surface relative to the tibial plateau and therefore reflect changes in the functional relationship between the patella and the tibial plateau, the IS index is primarily determined by the ratio of patellar tendon length to patellar length. Consequently, IS may be less sensitive to subtle alterations in joint alignment occurring during the early postoperative period. This observation is consistent with the existing literature demonstrating that the BP and CD indices exhibit substantially stronger agreement with each other than either does with the IS index, further supporting the notion that these methods are not interchangeable. Because each index is based on different anatomical landmarks and reflects distinct aspects of patellofemoral anatomy and biomechanics, their relative sensitivity and clinical utility may vary according to the underlying pathology and clinical setting [30,36]. Another possible explanation relates to the biological behavior of the patellar tendon. Although rapid weight loss immediately reduces compressive forces acting across the knee, structural remodeling of tendon tissue is generally considered a slower process than changes in joint loading or lower-limb alignment. Therefore, the early postoperative period may be sufficient to induce measurable changes in patellofemoral alignment, as reflected by BP and CD, while remaining too short for appreciable alterations in patellar tendon length, resulting in relatively stable IS values. Consistent with this interpretation, multivariable regression analyses demonstrated that baseline IS, but not BMI reduction, independently predicted postoperative ΔIS, suggesting that tendon-related adaptation may be influenced more by intrinsic anatomical characteristics than by the magnitude of weight loss itself. This hypothesis is further supported by the absence of significant correlations between ΔIS and either ΔBP or ΔCD, suggesting that tendon-related and joint-related adaptations may occur through partially distinct biomechanical pathways. From a clinical perspective, these findings also highlight that the three commonly used patellar height indices should not be regarded as interchangeable when evaluating early biomechanical adaptation after bariatric surgery. Although IS has traditionally been considered one of the most reproducible and reliable methods for assessing patellar height [31], the present findings suggest that BP and CD may be more sensitive to early alterations in patellofemoral alignment associated with rapid mechanical unloading. Whether the IS index becomes responsive during longer follow-up, as tendon remodeling and soft-tissue adaptation continue beyond the early postoperative period, remains to be determined.

An additional finding of the present study was the heterogeneous distribution of directional changes across the three patellar height indices (Figure 2). Although postoperative reductions predominated for both the BP and CD indices, approximately 40% of patients exhibited postoperative increases, while a small number showed no measurable change. In contrast, the IS index demonstrated an almost symmetrical distribution of increases and decreases despite the absence of a significant overall postoperative difference. These findings indicate that early radiographic adaptation following sleeve gastrectomy is not uniform across individuals. The bidirectional responses observed in a substantial proportion of patients suggest that, in addition to reduced mechanical loading, factors such as baseline anatomy, soft-tissue characteristics, and extensor mechanism geometry may contribute to the direction and magnitude of early radiographic adaptation. This interpretation is further supported by the multivariable regression analyses, which identified baseline patellar height indices as independent predictors of postoperative radiographic change.

Importantly, the primary objective of the present study was not to determine the prevalence of patella alta (superior patellar position) or patella baja (inferior patellar position) following bariatric surgery, but rather to characterize the subtle radiographic response of patellar height to rapid postoperative weight loss. Therefore, the analyses were based on continuous radiographic measurements rather than categorical classification using predefined cutoff values. This approach was considered appropriate because the reference ranges and diagnostic thresholds proposed for the commonly used patellar height indices vary according to the measurement method, study population, and clinical setting, and these indices are not considered interchangeable [30,36]. Furthermore, the use of predefined cutoff values may overlook subtle yet potentially meaningful radiographic changes that remain within the conventional normal range. We therefore believe that evaluating patellar height as a continuous variable provides greater sensitivity for detecting early biomechanical adaptation than a simple normal-versus-abnormal classification.

From a clinical perspective, although the observed radiographic changes were statistically significant, their absolute magnitude was modest and is unlikely to be clinically meaningful for individual patients in the early postoperative period. This observation is consistent with the current understanding that substantial weight loss after bariatric surgery produces rapid improvements in pain, mobility, and quality of life, whereas structural musculoskeletal remodeling may evolve more gradually over time [9,10,15,16]. Furthermore, it remains uncertain whether these differences exceed the inherent measurement variability of radiographic patellar height indices. Nevertheless, these findings suggest that the knee extensor mechanism undergoes early structural adaptation following rapid weight loss. Importantly, multivariable regression analyses demonstrated that baseline IS and BP indices independently predicted their respective postoperative changes, whereas BMI reduction was not an independent predictor. These findings suggest that, although rapid mechanical unloading may initiate early radiographic adaptation, baseline anatomical and structural characteristics remain among the principal determinants of postoperative patellar height. In other words, the early adaptive response appears to occur within the constraints of each individual’s pre-existing patellofemoral anatomy, indicating that the fundamental structural configuration of the extensor mechanism is largely preserved despite measurable postoperative changes. Improved recognition of these subtle biomechanical changes may enhance our understanding of postoperative musculoskeletal remodeling and provide a foundation for future studies investigating their relationship with anterior knee pain, patellofemoral function, and long-term knee health. Future studies incorporating repeated reliability measurements and longer-term follow-up are warranted to determine whether these early radiographic adaptations exceed measurement variability, remain stable, progress with continued weight loss, or eventually translate into clinically meaningful changes in knee function and patellofemoral biomechanics.

### 4.1. Limitations

The present study has several limitations. First, although all patients were followed prospectively according to a standardized institutional protocol, the study was designed as a single-center retrospective analysis and is therefore subject to the inherent limitations of retrospective research. Second, although several radiographic changes reached statistical significance, their absolute numerical magnitude was modest, and most of the observed correlations were weak to moderate in strength. Therefore, although the observed associations should be interpreted with appropriate caution given their modest effect sizes, they provide novel evidence regarding early postoperative adaptation of the knee extensor mechanism and establish a foundation for future longitudinal and biomechanical investigations. Third, potentially important patient-related factors that may influence musculoskeletal adaptation were not systematically recorded or analyzed. These include baseline metabolic comorbidities (e.g., diabetes mellitus and inflammatory rheumatologic disorders) and chronic pharmacological therapies that may affect connective tissue metabolism and collagen homeostasis (e.g., corticosteroids and nonsteroidal anti-inflammatory drugs). Such factors may influence tendon properties, connective tissue elasticity, neuromuscular function, and standing knee posture, and therefore could have affected the observed radiographic measurements. Fourth, radiographic assessment was limited to conventional lateral radiographs, and advanced imaging modalities such as magnetic resonance imaging were not available to evaluate soft-tissue structures of the extensor mechanism. Fifth, patellar height represents only one component of patellofemoral biomechanics, and additional parameters such as patellar tilt, trochlear morphology, patellar congruence, and tibial tubercle position were not assessed. Finally, the study intentionally included only consecutive patients undergoing primary sleeve gastrectomy at a single specialized bariatric center to ensure a homogeneous study population and standardized follow-up. Although this design strengthened internal validity, it may limit the external validity and generalizability of the findings to other bariatric procedures, healthcare settings, or patient populations. Additionally, since pharmacological weight-loss therapies, such as GLP-1 receptor agonists, were not evaluated, the present findings also cannot be generalized to patients achieving comparable weight reduction through nonsurgical treatment strategies. Multicenter studies including different bariatric techniques, pharmacological weight-loss interventions, and longer follow-up periods are warranted to externally validate the present findings.

### 4.2. Strengths

Nevertheless, this study also has important strengths. To our knowledge, this is the first study to investigate the relationship between rapid postoperative mechanical unloading and radiographic patellar height. The consecutive inclusion of patients, the prospective standardized follow-up, the use of a single experienced bariatric surgeon employing a uniform surgical technique, the standardized radiographic protocol, the consensus-based measurements performed by an experienced orthopedic review panel, and the incorporation of multivariable regression analyses to identify independent predictors of postoperative radiographic adaptation all enhance the methodological consistency and internal validity of the study.

## 5. Conclusions

In conclusion, the present findings suggest that rapid postoperative mechanical unloading following sleeve gastrectomy is accompanied by early radiographic adaptation of the patellofemoral joint. Although the magnitude of postoperative changes (Δ values) was not independently associated with the extent of weight loss or BMI reduction, multivariable regression analyses demonstrated that baseline patellar height indices independently predicted postoperative radiographic adaptation. These findings suggest that early postoperative changes occur within the constraints of an individual’s preexisting patellofemoral anatomy, indicating that baseline anatomical characteristics may influence early adaptation more than the magnitude of weight loss itself. Clinically, these findings reinforce the importance of an individualized postoperative musculoskeletal evaluation, recognizing that early biomechanical adaptation may vary substantially between patients despite comparable weight loss. Further longitudinal studies incorporating comprehensive biomechanical and functional assessments are warranted to determine the clinical relevance and long-term evolution of these early radiographic changes.

## Figures and Tables

**Table 1:** Changes in anthropometric characteristics and radiographic patellar height indices following sleeve gastrectomy.

Parameter		Median (Interquartile Range) (Minimum–Maximum Range)
Age (years)		33 (18) (18–63)
Gender	Female	123 (79.4%)
	Male	32 (20.6%)
Patient Height (m)		1.6 (0.13) (1.45–1.86)
Patient Weight (kg)	Preoperative	108.8 (25.8) (85.6–169)
	Postoperative	93 (24) (67–137.4)
	*p*-value	<0.001
	Δkg	17.9 (5.2) (8.8–32.4)
Body Mass Index (kg/m^2^)	Preoperative	41.98 (6.34) (35.08–57.75)
	Postoperative	35.3 (6.37) (26.64–50.09)
	*p*-value	<0.001
	ΔBMI	6.74 (1.92) (3.48–11.9)
Insall–Salvati Index	Preoperative	1.13 (0.25) (0.51–1.48)
	Postoperative	1.11 (0.22) (0.44–2.51)
	*p*-value	0.314
	ΔIS	0 (0.18) (−1.29–0.43)
Blackburne–Peel Index	Preoperative	0.94 (0.26) (0.48–1.41)
	Postoperative	0.9 (0.23) (0.37–2.78)
	*p*-value	0.004
	ΔBP	0.06 (0.17) (−2.01–0.54)
Caton–Deschamps Index	Preoperative	1.06 (0.2) (0.68–1.89)
	Postoperative	1.04 (0.32) (0.74–2.39)
	*p*-value	0.039
	ΔCD	0.06 (0.24) (−0.44–0.52)

Categorical variables are presented as n (%). Continuous variables are presented as median (interquartile range [IQR]; minimum–maximum). *p* values represent the statistical significance of the comparison between preoperative and postoperative measurements. Δ values were calculated as the preoperative value minus the corresponding postoperative value. BMI, body mass index; IS, Insall–Salvati index; BP, Blackburne–Peel index; CD, Caton–Deschamps index.

**Table 2:** Correlation between changes in body weight, body mass index, and radiographic patellar height indices following sleeve gastrectomy.

	Insall–Salvati Index		Blackburne–Peel Index		Caton–Deschamps Index	
	Postop	ΔIS	Postop	ΔBP	Postop	ΔCD
Age	0.793	0.004 (r = −0.231)	0.560	0.748	0.934	0.372
Patient Height	0.779	0.038 (r = 0.167)	0.186	0.585	0.675	0.512
Preoperative Weight	0.702	0.583	0.006 (r = 0.221)	0.832	0.496	0.191
Preoperative BMI	0.846	0.788	0.007 (r = 0.214)	0.238	0.342	0.339
Postoperative Weight	0.844	0.923	0.009 (r = 0.208)	0.892	0.660	0.252
Postoperative BMI	0.924	0.309	0.005 (r = 0.223)	0.222	0.520	0.426
ΔWeight	0.101	0.018 (r = 0.190)	0.755	0.309	0.341	0.153
ΔBMI	0.091	0.022 (r = 0.184)	0.713	0.636	0.409	0.200
Preoperative Insall–Salvati Index	<0.001 (r = 0.803)	0.326	<0.001 (r = 0.378)	0.139	0.011 (r = 0.203)	0.841
Preoperative Blackburne–Peel Index	<0.001 (r = 0.396)	0.328	<0.001 (r = 0.508)	0.010 (r = 0.207)	<0.001 (r = 0.302)	0.031 (r = 0.173)
Preoperative Caton–Deschamps Index	0.012 (r = 0.201)	0.108	<0.001 (r = 0.372)	0.112	<0.001 (r = 0.402)	<0.001 (r = 0.329)
Postoperative Insall–Salvati Index	N/A	<0.001 (r = −0.487)	<0.001 (r = 0.286)	0.738	0.078	0.849
Postoperative Blackburne–Peel Index	<0.001 (r = 0.286)	0.781	N/A	<0.001 (r = −0.673)	<0.001 (r = 0.744)	<0.001 (r = −0.673)
Postoperative Caton–Deschamps Index	0.078	0.791	<0.001 (r = 0.744)	<0.001 (r = −0.489)	N/A	<0.001 (r = −0.669)
ΔIS	<0.001 (r = −0.487)	N/A	0.781	0.170	0.791	0.170
ΔBP	0.738	0.292	<0.001 (r = −0.673)	N/A	<0.001 (r = −0.489)	<0.001 (r = 0.541)
ΔCD	0.849	0.170	<0.001 (r = −0.416)	<0.001 (r = 0.541)	<0.001 (r = −0.669)	N/A

Postop, postoperative; BMI, body mass index; IS, Insall–Salvati index; BP, Blackburne–Peel index; CD, Caton–Deschamps index; Δ, preoperative value minus the corresponding postoperative value; N/A, not applicable. *p* values are presented for all correlation analyses. Spearman’s correlation coefficients (r) are reported only for statistically significant correlations (*p* < 0.050) to improve table readability.

**Table 3:** Multiple linear regression analyses evaluating independent predictors of postoperative changes in patellar height indices after adjustment for age, sex, preoperative BMI, and BMI reduction.

Predictor	ΔIS β	*p*	ΔBP β	*p*	ΔCD β	*p*
Age	−0.157	0.055	−0.070	0.387	−0.104	0.208
Sex	0.004	0.966	0.071	0.400	0.126	0.143
Preoperative BMI	−0.074	0.396	−0.133	0.120	−0.064	0.457
ΔBMI	0.077	0.390	0.093	0.283	0.078	0.376
Baseline IS index	0.216	0.013	N/A	N/A	N/A	N/A
Baseline BP index	N/A	N/A	0.257	0.002	N/A	N/A
Baseline CD index	N/A	N/A	N/A	N/A	0.137	0.094
Adjusted R^2^	0.059		0.061		0.028	
Overall model (*p*)	0.015		0.013		0.099	

N/A, not applicable; BMI, body mass index; β, standardized regression coefficient; Δ, preoperative value minus the corresponding postoperative value; IS, Insall–Salvati index; BP, Blackburne–Peel index; CD, Caton–Deschamps index. Adjusted R^2^ indicates the proportion of variance explained by each multivariable regression model after adjustment for the number of predictors.

## Data Availability

The datasets generated and/or analyzed during the current study are stored in a private repository and are not publicly available due to ethical considerations, the protection of patients’ personal health data in accordance with applicable national data protection regulations, and the requirements of the Turkish Ministry of Health. However, the datasets may be made available from the corresponding author upon reasonable request and with approval from the relevant institutional authorities.

## Supplementary Materials

The following supporting information can be downloaded at: https://www.mdpi.com/article/10.3390/jcm15187137/s1. Supplementary File S1: STROBE Checklist.

## Abbreviations

The following abbreviations are used in this manuscript:

BMI	Body Mass Index
IS	Insall–Salvati Index
BP	Blackburne–Peel Index
CD	Caton–Deschamps Index
PACS	Picture Archiving and Communication System
IQR	Interquartile range
*p*	Statistical significance value
r	Spearman’s rank correlation coefficient
STROBE	Strengthening the Reporting of Observational Studies in Epidemiology

## References

[1] Ruseva A., Michalak W., Zhao Z., Fabricatore A., Hartaigh B.Ó., Umashanker D. (2024). Semaglutide 2.4 mg clinical outcomes in patients with obesity or overweight in a real-world setting: A 6-month retrospective study in the United States (SCOPE). Obes. Sci. Pract..

[2] Ruseva A., Dabbous F., Ding N., Fabricatore A., Huse S., Michalak W., Nordstrom B., Hartaigh B.Ó., Zhao Z., Umashanker D. (2025). Semaglutide 2.4 mg long-term clinical outcomes in patients with obesity or overweight: A real-world retrospective cohort study in the United States (SCOPE 12 months). Postgrad. Med..

[3] Toliver J.C., Divino V., Ng C.D., Wang J. (2025). Real-World Weight Loss Among Patients Initiating Semaglutide 2.4 mg and Enrolled in WeGoTogether, a Digital Self-Support Application. Adv. Ther..

[4] Pampanelli S., Terzoni S., Pantanetti P., Di Loreto C., Mazzieri A., Cangelosi G., Alberti S., Badiali C., Grandone I., Migliarelli S. (2026). Flexible dose of semaglutide reduces early discontinuation while maintaining comparable outcomes in obese patients: FLEX-SEMA 2.4 mg, an Italian real-world study. Diabetes Obes. Metab..

[5] Ruseva A., Michalak W., Fabricatore A., Hartaigh B.Ó., Zhao Z., Umashanker D. (2025). Semaglutide 2.4 mg Clinical Outcomes in Patients with Obesity or Overweight: A Real-World Retrospective Comparative Cohort Study. Adv. Ther..

[6] Adouni M., Aydelik H., Faisal T.R., Hajji R. (2024). The effect of body weight on the knee joint biomechanics based on subject-specific finite element-musculoskeletal approach. Sci. Rep..

[7] Eymard F., Aron-Wisnewsky J. (2024). Osteoarthritis in patients with obesity: The bariatric surgery impacts on its evolution. Jt. Bone Spine.

[8] Binvignat M., Sellam J., Berenbaum F., Felson D.T. (2024). The role of obesity and adipose tissue dysfunction in osteoarthritis pain. Nat. Rev. Rheumatol..

[9] Lehtovirta S., Kemppainen A., Haapea M., Nevalainen M., Lammentausta E., Kyllönen E., Koivukangas V., Lehenkari P., Karppinen J., Casula V. (2024). Effects of Bariatric Surgery on Knee Articular Cartilage and Osteoarthritis Symptoms-A 12-Month Follow-Up Using T2 Relaxation Time and WOMAC Osteoarthritis Index. J. Magn. Reson. Imaging.

[10] Alqahtani M.M.M., Raposo A., Alshalaan A.M.S., Asiri S.M.S., Alfaifi J.S.Y., Alqahtani S.M.S., Shawabkeh R.A.S., Abonukhaa A.A.S., Ahmed S.A.E., Asiri M.H.A. (2026). Effect of Bariatric Surgery on Osteoarthritis-Related Pain and Function: A Systematic Review and Meta-Analysis. World J. Surg..

[11] Alyafei A.A., AlMukhaini S.K., Ma Hussein A., Al Abdulla S.T. (2025). The Impact of Sleeve Gastrectomy Combined with Lifestyle Interventions on Anthropometric and Health Outcomes in Adults: A Systematic Review and Meta-Analysis. Cureus.

[12] Amorim-Cruz F., Fernandes Lopes D., Sousa-Pinto B., Santos-Sousa H. (2025). Efficacy and Safety of Fundoplication Sleeve Gastrectomy in Obesity and GERD: A Systematic Review and Meta-Analysis. J. Clin. Med..

[13] Özen G.Ü., Büyükkasap Ç., Dursun B., Akyol A. (2026). Longitudinal Changes in Serum Procalcitonin After Bariatric Surgery and Their Associations with Anthropometric, Metabolic, and Inflammatory Parameters. J. Clin. Med..

[14] Khormi K.A., Mumena W.A., Salman A.K.M., Faden A.A., Hafiz M.S., Kutbi H.A. (2026). Long-Term Weight Loss Outcomes Following Sleeve Gastrectomy and Their Association with Diet Quality, Postoperative Complications, and Sociodemographic Factors: A Retrospective Cohort Study in Jeddah, Saudi Arabia. J. Clin. Med..

[15] Vincent H.K., Ben-David K., Cendan J., Vincent K.R., Lamb K.M., Stevenson A. (2010). Effects of bariatric surgery on joint pain: A review of emerging evidence. Surg. Obes. Relat. Dis..

[16] Rebibo L., Verhaeghe P., Tasseel-Ponche S., Cosse C., Maréchal V., Dhahri A., Doutrellot P.L., Regimbeau J.M. (2016). Does sleeve gastrectomy improve the gait parameters of obese patients?. Surg. Obes. Relat. Dis..

[17] Guan L., Zhang W., Wang H., Zhang Y., Sun J., Lu J. (2026). Effects of Mechanical Loading on the Structure and Function of the Achilles Tendon: From Homeostatic Adaptation to Pathological Degeneration. J. Funct. Morphol. Kinesiol..

[18] Erdoğan E., Akay Ö., Koncalıoğlu B., Güler M., Gencer B. (2026). Differential Radiographic Response of Sagittal Foot Alignment to Early Weight Loss Following Sleeve Gastrectomy. Medicina.

[19] Türkoğlu F., Sarı S., Gencer E.N., Sağlam T., Doğan Ö. (2026). Early Weight Loss After Sleeve Gastrectomy Selectively Improves Intermetatarsal Angle Without Affecting Hallux Valgus Angle. J. Clin. Med..

[20] Doğan Ö., Çulcu A., Doğan İ.S. (2023). Patellar height changes after treatment of tibia plateau fractures: A radiological analysis. Saudi Med. J..

[21] Igoumenou V.G., Dimopoulos L., Mavrogenis A.F. (2019). Patellar Height Assessment Methods: An Update. JBJS Rev..

[22] Doğan Ö., Gencer B., Süer Doğan İ. (2023). The effectiveness of anterior cruciate ligament reconstruction on the patellofemoral stability and patellar height. Arch. Curr. Med. Res..

[23] Thompson P., Metcalfe A.J. (2019). Current concepts in the surgical management of patellar instability. Knee.

[24] Salem K.H., Sheth M.R. (2021). Variables affecting patellar height in patients undergoing primary total knee replacement. Int. Orthop..

[25] Gencer B., Yiğit A., Çamoğlu C., Çulcu A., Dogan O. (2023). Can Anterior Knee Pain Be Explained by Patella Position After Infrapatellar Tibia Intramedullary Nailing?. Cureus.

[26] Carissimi M., Sautet P., Charre D., Hanak L., Ollivier M., Micicoi G. (2021). Patellar height is not modified after isolated open-wedge high tibial osteotomy without change in posterior tibial slope. Orthop. Traumatol. Surg. Res..

[27] Vampertzis T., Barmpagianni C., Nitis G., Papastergiou S. (2020). A study of the possible effect of abnormal patella height on meniscal tears. J. Orthop..

[28] von Elm E., Altman D.G., Egger M., Pocock S.J., Gøtzsche P.C., Vandenbroucke J.P., STROBE Initiative (2008). The Strengthening the Reporting of Observational Studies in Epidemiology (STROBE) statement: Guidelines for reporting observational studies. J. Clin. Epidemiol..

[29] Elmaleh-Sachs A., Schwartz J.L., Bramante C.T., Nicklas J.M., Gudzune K.A., Jay M. (2023). Obesity Management in Adults: A Review. JAMA.

[30] Seil R., Müller B., Georg T., Kohn D., Rupp S. (2000). Reliability and interobserver variability in radiological patellar height ratios. Knee Surg. Sports Traumatol. Arthrosc..

[31] Verhulst F.V., van Sambeeck J.D.P., Olthuis G.S., van der Ree J., Koëter S. (2020). Patellar height measurements: Insall-Salvati ratio is most reliable method. Knee Surg. Sports Traumatol. Arthrosc..

[32] Picken S., Summers H., Al-Dadah O. (2022). Inter- and intra-observer reliability of patellar height measurements in patients with and without patellar instability on plain radiographs and magnetic resonance imaging. Skelet. Radiol..

[33] Rocha C.C.D., Pinheiro R.S., Pavan I.A., Saito R.Y., Costa P.P.D., Cunha H.E.F. (2023). Effects of bariatric surgery on knee joint pain. Acta Ortop. Bras..

[34] Li J.S., Tsai T.Y., Clancy M.M., Li G., Lewis C.L., Felson D.T. (2019). Weight loss changed gait kinematics in individuals with obesity and knee pain. Gait Posture.

[35] Bin S.I., Kim H.J., Ahn H.S., Rim D.S., Lee D.H. (2016). Changes in Patellar Height After Opening Wedge and Closing Wedge High Tibial Osteotomy: A Meta-analysis. Arthroscopy.

[36] Hunter C.D.R., Khalil A.Z., Rosenthal R.M., Metz A.K., Featherall J., Ernat J.J., Aoki S.K. (2024). Common radiographic indices used to measure patellar height do not consistently identify patella alta and lack interchangeability between measurements. Knee Surg. Sports Traumatol. Arthrosc..

