# Supplementary material for: Radiographic Changes in Patellar Height Following Rapid Weight Loss After Sleeve Gastrectomy: A Retrospective Observational Study
Source: J Clin Med. 2026 Sep 14;15(18):7137. doi: 10.3390/jcm15187137 (PMC13607650; doi:10.3390/jcm15187137)
Supplement: Supplementary file 1 [file jcm-15-07137-s001.zip › jcm-4533630-supplementary.pdf]

STROBE Statement—checklist of items that should be included in reports of observational studies

	Item No.	Recommendation	Page No.	Relevant text from manuscript
**Title and abstract**	1	(*a*) Indicate the study's design with a commonly used term in the title or the abstract	1	Title and Abstract
		(*b*) Provide in the abstract an informative and balanced summary of what was done and what was found	1	Abstract
**Introduction**				
Background/rationale	2	Explain the scientific background and rationale for the investigation being reported	2-3	Introduction – 2nd and 3rd paragraphs
Objectives	3	State specific objectives, including any prespecified hypotheses	3	Introduction – last paragraph
**Methods**				
Study design	4	Present key elements of study design early in the paper	3-4	Methods – *"Study Design and Ethical Approval"* and "*Patient Selection*"
Setting	5	Describe the setting, locations, and relevant dates, including periods of recruitment, exposure, follow-up, and data collection	3-4	Methods – *"Study Design and Ethical Approval"* and "*Patient Selection*"
Participants	6	(*a*) *Cohort study*—Give the eligibility criteria, and the sources and methods of selection of participants. Describe methods of follow-up<br>*Case-control study*—Give the eligibility criteria, and the sources and methods of case ascertainment and control selection. Give the rationale for the choice of cases and controls<br>*Cross-sectional study*—Give the eligibility criteria, and the sources and methods of selection of participants	3-4	Methods – *"Study Design and Ethical Approval", and "Patient Selection"* (eligibility criteria, consecutive patient inclusion, and standardized 4-month follow-up)
		(*b*) *Cohort study*—For matched studies, give matching criteria and number of exposed and unexposed<br>*Case-control study*—For matched studies, give matching criteria and the number of controls per case	N/A	N/A
Variables	7	Clearly define all outcomes, exposures, predictors, potential confounders, and effect modifiers. Give diagnostic criteria, if applicable	4-6	Methods – *"Clinical and Demographic Data Collection", "Radiographic Assessment", "Observer Assessment"* and "*Primary and Secondary Outcomes*"
Data sources/ measurement	8*	For each variable of interest, give sources of data and details of methods of assessment (measurement). Describe comparability of assessment methods if there is more than one group	4-5	Methods – *"Clinical and Demographic Data Collection", "Radiographic Assessment",* and *"Observer Assessment"*
Bias	9	Describe any efforts to address potential sources of bias	3-5	Methods – "*Patient Selection*", "*Radiographic Assessment*", and "*Observer Assessment*" (consecutive patient inclusion, predefined eligibility criteria, standardized imaging protocol, blinded consensus assessment, .and a priori right-knee selection to avoid statistical dependence between paired observations)
Study size	10	Explain how the study size was arrived at	3-4	Methods – "*Patient Selection*" (all consecutive eligible patients during the study period were included; no additional sampling performed)

Quantitative variables	11	Explain how quantitative variables were handled in the analyses. If applicable, describe which groupings were chosen and why	4 and 6	Methods – "*Clinical and Demographic Data Collection*", and "*Statistical Analysis*" (continuous variables were analysed as continuous measures; Δ values were calculated as preoperative minus postoperative values; no categorization of quantitative variables was performed)
Statistical methods	12	(*a*) Describe all statistical methods, including those used to control for confounding	6	Materials and Methods – *"Statistical Analysis*" section
		(*b*) Describe any methods used to examine subgroups and interactions	N/A	N/A
		(*c*) Explain how missing data were addressed	3-4	Methods – "*Patient Selection*" (only patients with complete demographic, anthropometric, and radiographic data were included; patients with incomplete postoperative follow-up were excluded)
		(*d*) *Cohort study*—If applicable, explain how loss to follow-up was addressed<br>*Case-control study*—If applicable, explain how matching of cases and controls was addressed<br>*Cross-sectional study*—If applicable, describe analytical methods taking account of sampling strategy	N/A	N/A
		(*e*) Describe any sensitivity analyses	N/A	N/A(no sensitivity analyses were performed).
**Results**				
Participants	13*	(a) Report numbers of individuals at each stage of study—eg numbers potentially eligible, examined for eligibility, confirmed eligible, included in the study, completing follow-up, and analysed	7	Figure 1. Study flow diagram
		(b) Give reasons for non-participation at each stage	7	Figure 1. Study flow diagram
		(c) Consider use of a flow diagram	7	Figure 1
Descriptive data	14*	(a) Give characteristics of study participants (eg demographic, clinical, social) and information on exposures and potential confounders	6-8	Results – "*Patient Characteristics and Radiographic Findings"*
		(b) Indicate number of participants with missing data for each variable of interest	3-4,6-7	Methods – "*Patient Selection*", and Results (No missing data were present for the variables included in the analyses. Only patients with complete demographic, anthropometric, and radiographic data were included)
		(c) *Cohort study*—Summarise follow-up time (eg, average and total amount)	3-4	Methods – "*Patient Selection*" (Standardized follow-up at the routine fourth postoperative month for all included participants)
Outcome data	15*	*Cohort study*—Report numbers of outcome events or summary measures over time	6-10	Results, Tables (Changes in IS, BP, and CD indices between the preoperative assessment and the routine fourth postoperative month)
		Case-control study—Report numbers in each exposure category, or summary measures of exposure	N/A	N/A

		Cross-sectional study—Report numbers of outcome events or summary measures	N/A	N/A
Main results	16	(*a*) Give unadjusted estimates and, if applicable, confounder-adjusted estimates and their precision (eg, 95% confidence interval). Make clear which confounders were adjusted for and why they were included	6-10	Results – Correlation analyses, multivariable regression analyses, and Tables 2–4.
		(*b*) Report category boundaries when continuous variables were categorized	N/A	N/A
		(*c*) If relevant, consider translating estimates of relative risk into absolute risk for a meaningful time period	N/A	N/A
Other analyses	17	Report other analyses done—eg analyses of subgroups and interactions, and sensitivity analyses	6-10	Discussion of additional correlation and multivariable regression analyses
**Discussion**				
Key results	18	Summarise key results with reference to study objectives	10-11	Discussion – first and second paragraphs
Limitations	19	Discuss limitations of the study, taking into account sources of potential bias or imprecision. Discuss both direction and magnitude of any potential bias	14	Discussion – "Limitations"
Interpretation	20	Give a cautious overall interpretation of results considering objectives, limitations, multiplicity of analyses, results from similar studies, and other relevant evidence	10-14	Discussion – Interpretation of findings and clinical implications
Generalisability	21	Discuss the generalisability (external validity) of the study results	13-14	Discussion – Generalisability paragraph
**Other information**				
Funding	22	Give the source of funding and the role of the funders for the present study and, if applicable, for the original study on which the present article is based	15	Funding Statement
